# Socio-economic, governance and health indicators shaping antimicrobial resistance: an ecological analysis of 30 european countries

**DOI:** 10.1186/s12992-023-00913-0

**Published:** 2023-02-24

**Authors:** Andrea Maugeri, Martina Barchitta, Federico Puglisi, Antonella Agodi

**Affiliations:** 1 Department of Medical and Surgical Sciences and Advanced Technologies “GF Ingrassia”, University of Catania, Via S. Sofia 87, 95123 Catania, Italy; 2 Department of Economics and Business, University of Catania, Corso Italia 55, 95129 Catania, Italy

**Keywords:** Antibiotic, Antimicrobial, Resistance, Social factors, Economic, Governance, Corruption, Health expenditure

## Abstract

**Background:**

Previous evidence shows that antibiotic use and antimicrobial resistance (AMR) spread are not always perfectly correlated within and between countries. We conducted an ecological analysis to evaluate how demographic, economic, governance, health, and freedom characteristics of 30 European countries contribute to antibiotic consumption and AMR.

**Methods:**

Using three sources of data (World Bank DataBank, ECDC atlas, and the ESAC-Net database), we created a dataset of: 22 indicators of demographics, health, economic, governance, and freedom; AMR proportions for 25 combinations of pathogens and antibiotics; consumption of antibiotics in the community. We also computed five indexes of demographic, health, economic, governance, and freedom, and an aggregate AMR measure. Relationships between indexes, antibiotic consumption, and AMR proportions were explored using bivariate, multivariable, multivariate, and mediation analyses.

**Results:**

Multivariate analysis identified three clusters of countries that mainly differed for demographic, health, governance, and freedom indexes. AMR proportion was lower in countries with better indexes (*p* < 0.001), but not necessarily with lower antibiotic consumption. In multivariable models including all five indexes, an increase in the governance index resulted in significant decreases of overall antibiotic consumption (*p* < 0.001) and AMR proportion (*p* = 0.006). Mediation analysis showed that the governance index had an indirect effect on AMR via reducing antibiotic consumption, which accounted only for 31.5% of the total effect.

**Conclusions:**

These findings could be – at least partially – explained by the contagion theory, for which other factors contribute to high levels of AMR in countries with poor governance. As a result of this evidence, reducing antibiotic use alone is unlikely to solve the AMR problem, and more interventions are needed to increase governance efficiency at global level.

**Supplementary Information:**

The online version contains supplementary material available at 10.1186/s12992-023-00913-0.

## Background

Antimicrobial resistance (AMR) remains a serious public health concern, which imposes a large clinical and economic burden worldwide [1–3]. Although most of this burden is concentrated in low- and middle-income countries (LMICs), the numbers of resistant pathogens continue to increase also in many high-income countries (HICs) [4, 5]. Recent estimates from the European Union/European Economic Area (EU/EEA), in fact, show that more than 670,000 annual infections are due to AMR pathogens and nearly 33,000 people die as a direct consequence of AMR [6]. As fully discussed elsewhere [7], the general view is that AMR is almost entirely related to the volume of antibiotics used in the human and animal sectors and to the contagion (i.e., the spread of AMR pathogens and/or genetic elements of resistance in the environment). This idea, however, does not have the full support of observations showing higher AMR proportion in several LMICs, in which antibiotic consumption is much lower than in HICs [4, 5]. In fact, previous evidence shows how antibiotic use and AMR spread are not always perfectly correlated within and between countries [8–12].

Observed disparities between countries may also be explained by social, governance and economic factors contributing to AMR. It is widely known that health outcomes are affected by some of the most common development indicators (e.g., quality of governance, health expenditures, gross domestic product – GDP – per capita, education, and community infrastructure) [7]. This might be true even in the case of AMR, which is influenced by numerous factors beyond antibiotic abuse. The study by Collignon and colleagues was a paving stone in this field, showing positive correlations of AMR proportions with poorer administrative governance, the ratio of private to public health expenditure, and warmer temperature. The Authors found strong and positive effects of infrastructure and administrative governance—particularly in terms of low corruption levels—in reducing AMR proportions when more factors were considered together. Contrary to what was expected, instead, antibiotic consumption was not significantly associated with AMR levels [13]. In their comment on this finding, Collignon and Beggs therefore emphasized that contagion is the main contributing factor of global disparities in AMR [7]. The spread of resistant pathogens is in fact a crucial problem in all healthcare settings, but also in the community. Based on the contagion hypothesis, AMR levels tend to be higher in LMICs, with poor infrastructure, poor community hygiene, weak administrative governance, and weak social commitment [7].

Inspired by the research by Collignon and colleagues, we developed the hypothesis that other factors are as important – or even more important than antibiotic abuse – to explain disparities in AMR proportions observed across European countries. Although countries consuming higher levels of antibiotics are also those reporting higher AMR levels, the response to any change in antibiotic use is relatively slow [7]. Indirectly, this indicates that other factors are also at play, adding to the complexity of the problem. For these reasons, the purpose of the present ecological analysis was to evaluate how demographic, economic, governance, health, and freedom factors of European countries contribute to antibiotic consumption and AMR. With this aim, we first evaluated the individual and multivariable relationships between these factors, antibiotic consumption, and AMR. For those factors exhibiting a dual relationship with antibiotic consumption and AMR, we hypothesized and investigated a potential mediating effect. Finally, a multivariate analysis was conducted to explore the complex relationships between factors characterizing European countries, antibiotic consumption, and AMR.

## Methods

### Data sources

We compiled a dataset for 30 European countries (all 27 countries of the European Union and three additional countries with available data: Iceland, Norway, and United Kingdom) on data related to 2019 or, if this was not available, to the latest year no earlier than 2016. Data for the subsequent years was not used to prevent the analysis being affected by the COVID-19 pandemic. The dataset consisted of: twenty World Bank indicators of demographics, health, economic, and governance [14]; political rights and civil liberties scores from the Freedom House [15]; proportions of AMR for twenty-five combinations of pathogens and antibiotics from the European Centre for Disease Prevention and Control (ECDC) atlas [16]; community consumption of antibiotics for systemic use in general (J01), as well as of beta-lactam penicillins (J01C), other beta-lactam antibiotics (J01D), aminoglycosides (J01G), quinolones (J01M), and other antibiotics (J01X) from the ESAC-Net database [17] (antibiotic consumption was expressed as Defined Daily Dose [DDD] per 1,000 residents and per day).

Due to the multiple variables that contribute to antibiotic consumption and AMR, many of which are interrelated, we synthesized the data into aggregated indexes that capture the main probable influences. The aggregation procedure, borrowed from Collignon and colleagues [13], first rescaled individual indicators in order to make them comparable, and then constructed an average of them to arrive at aggregate indexes. The demographic index included seven indicators related to population density, land area and total population of countries, to fertility and population growth, and to population composition in terms of age and urbanization. The health index combined one of the most frequently used health status indicators (i.e., life expectancy at birth) with the general government expenditure and per capita total expenditure on health. The economic index was made of four indicators related to current GDP and its growth, which are widely used by economists to gauge the health of an economy. The governance index included indicators for six dimensions of governance (i.e., voice and accountability; political stability; government effectiveness; regulatory quality; rule of law; and control of corruption). The freedom index was computed by aggregating the political rights and the civil liberties scores. Each aggregate index was constructed by averaging the standardized values of indicators composing the index. For each country, indicators were standardized by subtracting the overall mean and then dividing by the overall standard deviation (SD).

In a similar way, we created a measure, termed aggregate AMR, to identify the relationships between AMR and indexes described earlier. The aggregate measure was computed as the average of resistance proportions for all the twenty-five combinations of pathogens and antibiotics. Prior to calculating the aggregate measure, missing values for country-specific AMR proportions were replaced with the average value obtained from countries with available data.

### Statistical analysis

Descriptive statistics were used to summarize the main features of European countries. Bivariate analyses were performed by the Spearman’s correlation and reported with the correlation coefficient ρ. Bonferroni correction was used to adjust for multiple comparisons. Linear regression models were further applied to identify aggregate indexes that most affected antibiotic use and resistance across European countries. The first model included the consumption of antibiotics for systemic use in the community as the dependent variable, and aggregate indexes described earlier as independent variables. The second model included the aggregate measure of AMR as the dependent variable, with aggregate indexes and antibiotic consumption as independent variables. According to the Shapiro–Wilk Test, all variables were first log-transformed to ensure their normality before being included in the models. All results were reported as β coefficients and their standard error (SE).

A potential mediating effect has been examined for indexes that exhibited linear relationships with both antibiotic consumption and AMR. In particular, a mediation analysis was carried out to evaluate whether antibiotic consumption mediated the relationships between the governance index and the aggregate measure of AMR. In general, the mediation analysis decomposes the total effect between the exposure and the outcome into a direct and an indirect effect through a mediator variable [18]. In particular, our analysis followed the procedure described by Preacher and Hayes [19]: the *path a* regressed the mediator (i.e., consumption of antibiotics for systemic use in the community) on the independent variable (i.e., governance the index); the *path b* regressed the dependent variable (i.e.. the aggregate AMR measure) on the mediator; the *path c’* regressed the dependent variable on the independent variable, adjusting for the effect of the mediator. All other aggregate indexes were included in the model as covariates. Bootstrapping with 5,000 resamples was used to calculate the bias-corrected and accelerated confidence intervals (CIs) for the indirect effect (a*b). The percentage mediated was expressed as the percentage of the total effect (path c) accounted for by the indirect effect (a*b).

Multivariate analysis was conducted using the K-means algorithm to reveal different clusters of European countries according to aggregate indexes described earlier. The best cluster solution was determined by the number of clusters that maximized the silhouette score [20]. Antibiotic consumption and resistance were compared across clusters using the Kruskal–Wallis test.

All statistical analyses were performed using the SPSS software (version 26.0, SPSS, Chicago, IL), with a significance level α of 0.05, unless otherwise indicated.

## Results

### Correlations between indicators, antibiotic consumption, and AMR

**Fig. 1:**
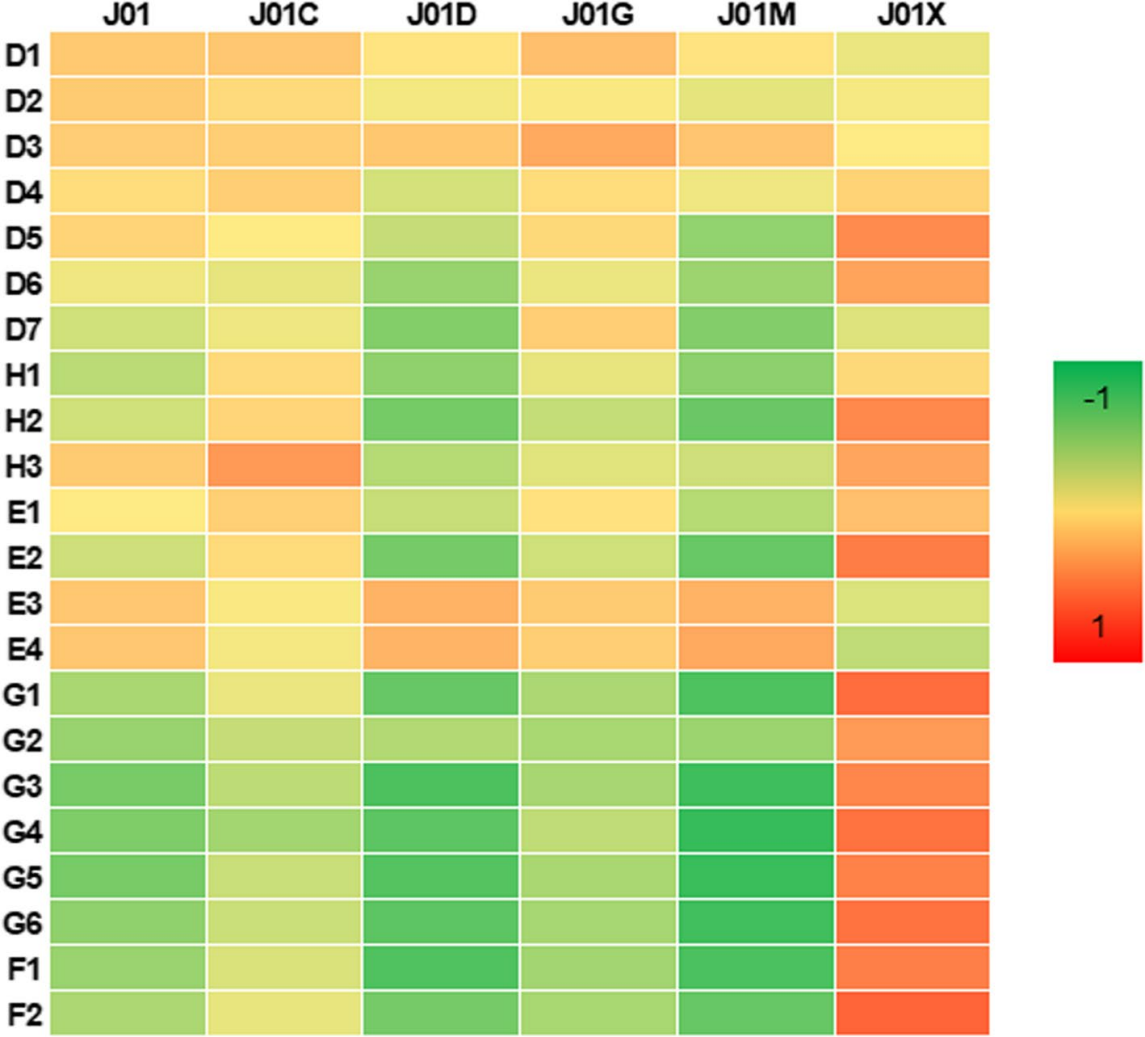
Heatmap of the Spearman correlation matrix between indicators and antibiotic consumptions. Correlation coefficients from -1 (in green) to 1 (in red)

Data on indicators and antibiotic consumption were available for each of the 30 European countries under consideration (Table 1 and Table S1, respectively). The median consumption of antibiotics for systemic use in the community was 17.4 DDD per 1,000 residents and per day (Interquartile Range [IQR] = 7.7). The heatmap of correlation analysis between indicators and antibiotic consumption is depicted in Fig. 1. Although no indicators correlated with the overall consumption of antibiotics in the community, indicators of governance were strongly and inversely correlated with the consumption of other beta-lactams (J01D) and quinolones (J01M) after Bonferroni correction. Similar correlations were evident for the political rights score (Table S2).

**Table 1:** Indicators of demographic, health, economic, governance, and freedom domains

Indicator	Abbreviation	Mean	Standard deviation	Minimum	Maximum	Median	IQR
**Demographic indicators**							
Total population	D1	17,337,495.0	23,344,767.2	360,563.0	83,092,962.0	7,927,840.5	15,232,512
Land area, km^2^	D2	156,916.3	156,509.4	320.0	547,557.0	86,890.0	25,6675.0
Population density, people per km^2^	D3	173.5	276.6	3.5	1,514.5	107.1	99.7
Annual population growth	D4	0.7	0.8	0.0	3.9	0.6	0.5
Population aged > 40	D5	0.5	0.0	0.4	0.5	0.5	0.1
Urban population, % of total population	D6	74.9	13.2	53.7	98.0	74.6	20.8
Fertility rate, total births per woman	D7	1.5	0.2	1.1	1.9	1.6	0.3
**Health indicators**							
Health expenditure, % of total GDP	H1	8.3	1.8	5.3	11.4	8.4	3.3
Health expenditure per capita, US$	H2	3,367.7	2,191.3	687.3	8,239.1	2,744.9	4,074.6
Life expectancy at birth, years	H3	80.3	2.7	74.9	83.5	81.2	4.2
**Economic indicators**							
GDP, billion US$	E1	629.3	966.6	15.08	3,860.0	250.5	489.5
GDP per capita, US$	E2	37,924.4	23,844.9	9,828.1	114,685.2	29,650.9	31,023.6
5-year average GDP growth	E3	3.2	1.8	0.9	10.1	3.1	2.2
5-year average GDP growth per capita	E4	2.8	1.8	0.7	8.9	2.6	2.7
**Governance indicators**							
Voice and accountability	G1	1.1	0.4	0.2	1.7	1.1	0.4
Political stability	G2	0.8	0.3	0.3	1.7	0.8	0.5
Government effectiveness	G3	1.1	0.5	0.3	1.9	1.1	0.9
Regulatory quality	G4	1.2	0.4	0.5	1.9	1.2	0.6
Rule of law	G5	1.2	0.6	0.0	2.0	1.1	1.2
Control of corruption	G6	1.1	0.8	0.0	2.2	0.8	1.5
**Freedom indicators**							
Political rights score	F1	37.2	2.8	26.0	40.0	38.0	3.0
Civil liberties score	F2	54.2	4.3	43.0	60.0	55.0	5.0

**Fig. 2:**
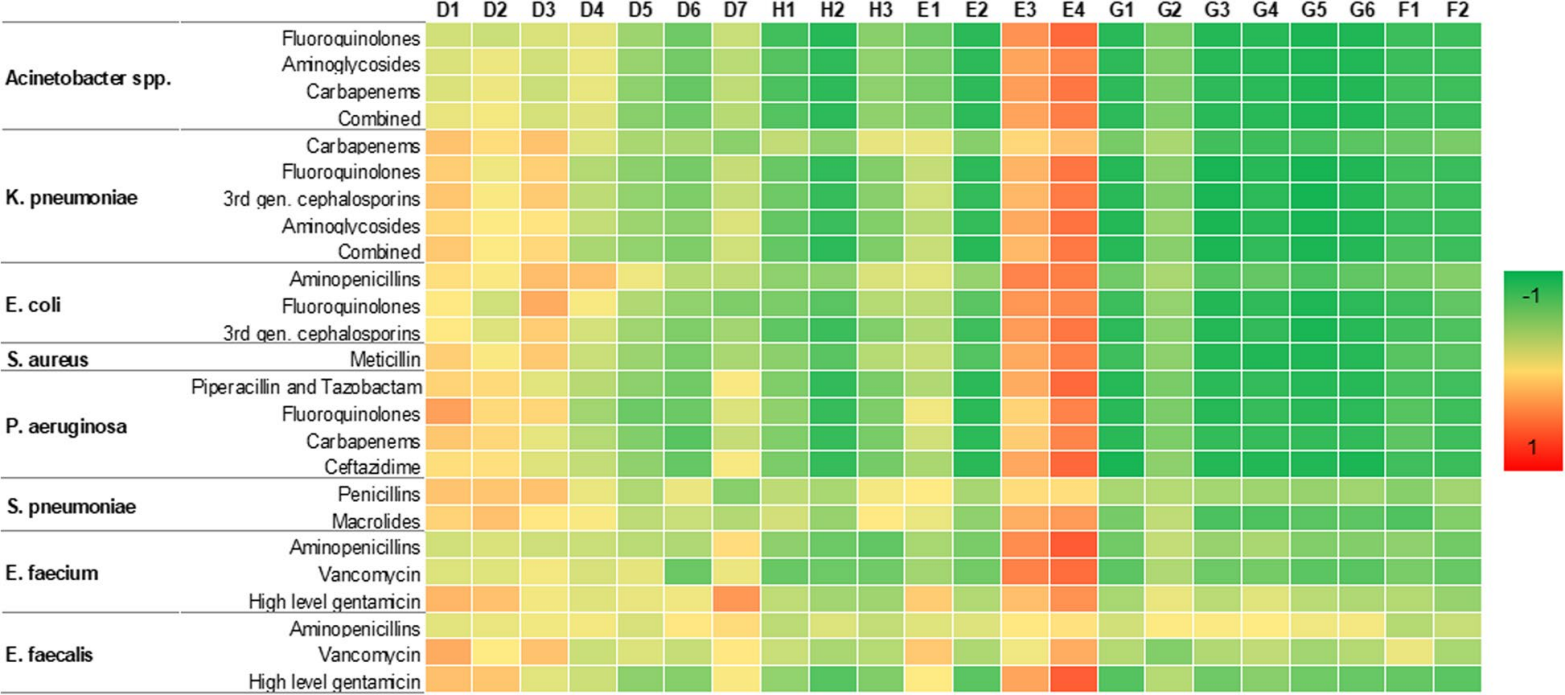
Heatmap of the Spearman correlation matrix between indicators and proportion of AMR for each combination between pathogen and antibiotic. Correlation coefficients from -1 (in green) to 1 (in red)

AMR proportions for specific combinations between pathogens and antibiotics were available for 26 countries or more (Table S3). The heatmap of correlation analysis between indicators and AMR proportions is depicted in Fig. 2. Correlations that were significant after Bonferroni correction are outlined below. Among indicators of health, the proportion of health expenditure was strongly and inversely correlated with the proportion of *Acinetobacter* spp. resistant to fluoroquinolones and carbapenems. Beyond that, health expenditure per capita was strongly and inversely correlated with the proportions of: *Acinetobacter* spp. and *P. aeruginosa* resistant to all classes of antibiotics under consideration; *K. pneumoniae* resistant to fluoroquinolones, third generation cephalosporins, and aminoglycosides; and *E. coli* resistant to third generation cephalosporins. Similar correlation coefficients were obtained for the GDP per capita, which was included among the economic indicators. With the exception of political stability, all indicators of governance (voice and accountability, government effectiveness, regulatory quality, rule of law, and control of corruption) were strongly and inversely correlated with the proportions of: *Acinetobacter* spp. and *P. aeruginosa* resistant to all classes of antibiotics under consideration; *K. pneumoniae* resistant to fluoroquinolones, third generation cephalosporins, and aminoglycosides; *E. coli* resistant to fluoroquinolones and third generation cephalosporins; and *S. aureus* resistant to methicillin. Government effectiveness and regulatory quality were also inversely correlated with the proportion of *S. pneumoniae* resistant to macrolides, whereas rule of law inversely also correlated with the proportion of *E. coli* resistant to aminopenicillins. A similar pattern of correlations was evident for indicators of freedom (political rights and civil liberties scores), which were strongly and inversely correlated with the proportion of: *Acinetobacter* spp. resistant to all classes of antibiotics under consideration; *K. pneumoniae* resistant to fluoroquinolones, third generation cephalosporins, and aminoglycosides; *E. coli* resistant to third generation cephalosporins; and *P. aeruginosa* resistant to piperacillin and tazobactam and ceftazidime. Political rights score was also inversely correlated with the proportions of *E. coli* resistant to fluoroquinolones and *S. pneumoniae* resistant to macrolides, whereas civil liberties score inversely correlated with the proportions of *P. aeruginosa* resistant to fluoroquinolones and carbapenems. Correlation coefficients for all the analyses described earlier are reported in the Table S4.

### Relationship between aggregate indexes, antibiotic consumption, and AMR

Because several indicators – which were also interrelated to each other – might affect antibiotic consumption and resistance, we also performed correlation and multivariable regression analyses using five aggregate indexes related to the demographic, health, economic, governance, and freedom domains. Levels of each index across European countries are depicted in the Figures S1-S5. After Bonferroni correction, the governance index was inversely correlated with the overall consumption of antibiotics in the community, as well as with the consumption of other beta-lactams (J01D) and quinolones (J01M); the health index was inversely correlated with the consumption of quinolones; and the freedom index was inversely correlated with the consumption of other beta-lactams and quinolones (Table S5). In the multivariable regression model including all aggregate indexes, an increase in the governance index resulted in significant decrease of overall antibiotic consumption in the community (Table 2). We also observed a marginally significant increase of overall antibiotic consumption associated with an increase in the freedom index (*p* = 0.054).

**Table 2:** Multivariable linear regression of the relationship between aggregate indexes and the consumption of antibiotics for systemic use in the community

Indexes	Beta	Standard Error	*p*-value
Demographic	0.480	0.510	0.356
Health	0.408	0.285	0.165
Economic	0.585	0.445	0.201
Governance	-1.242	0.270	< 0.001
Freedom	0.117	0.058	0.054
R^2^	0.510		

**Fig. 3:**
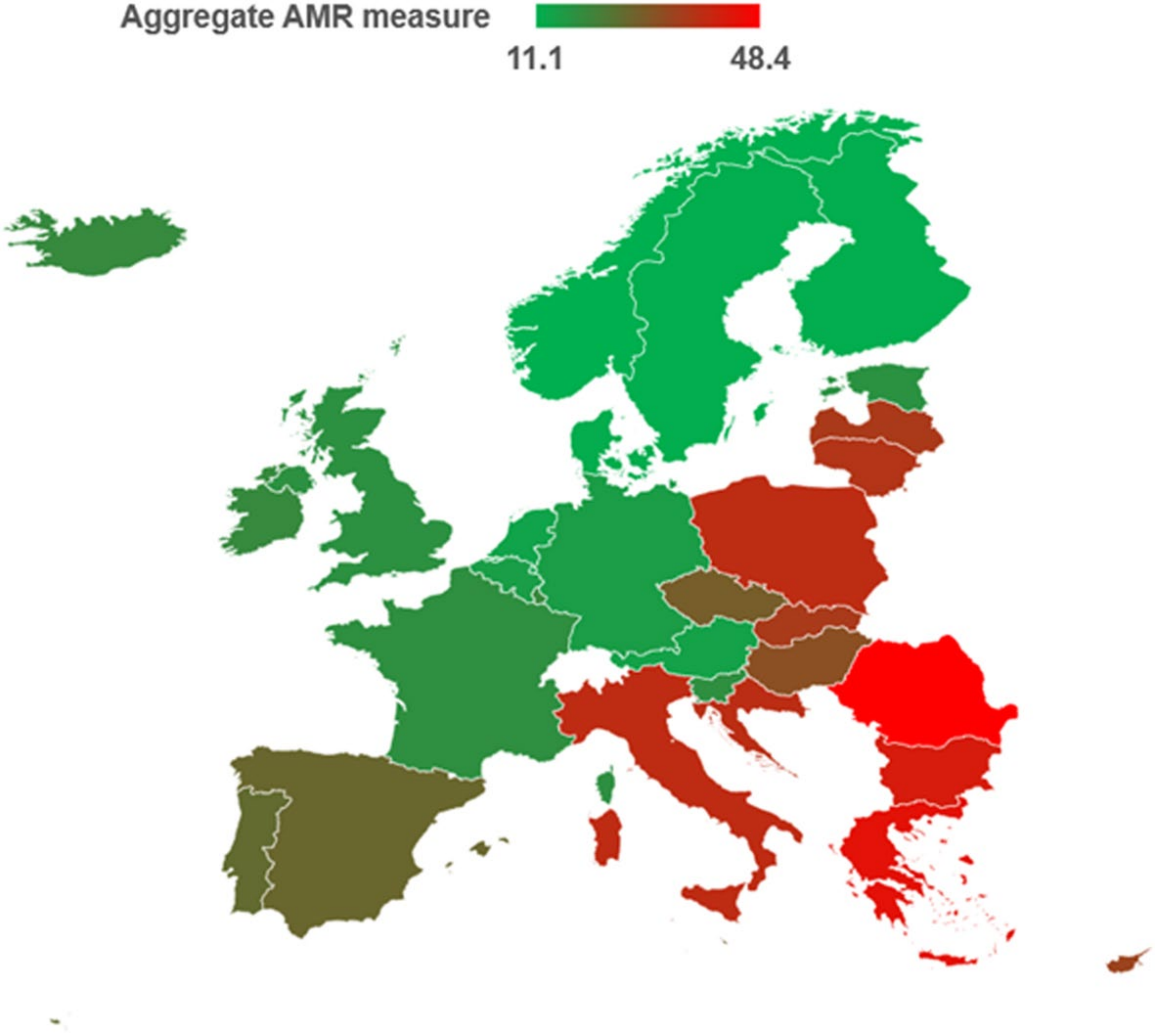
Aggregate AMR measure across European countries. Map shows levels of the aggregate AMR measure from low (in green) to high (in red). The aggregate measure was calculated as the average of AMR proportions for all the twenty-five combinations of pathogens and antibiotics

**Fig. 4:**
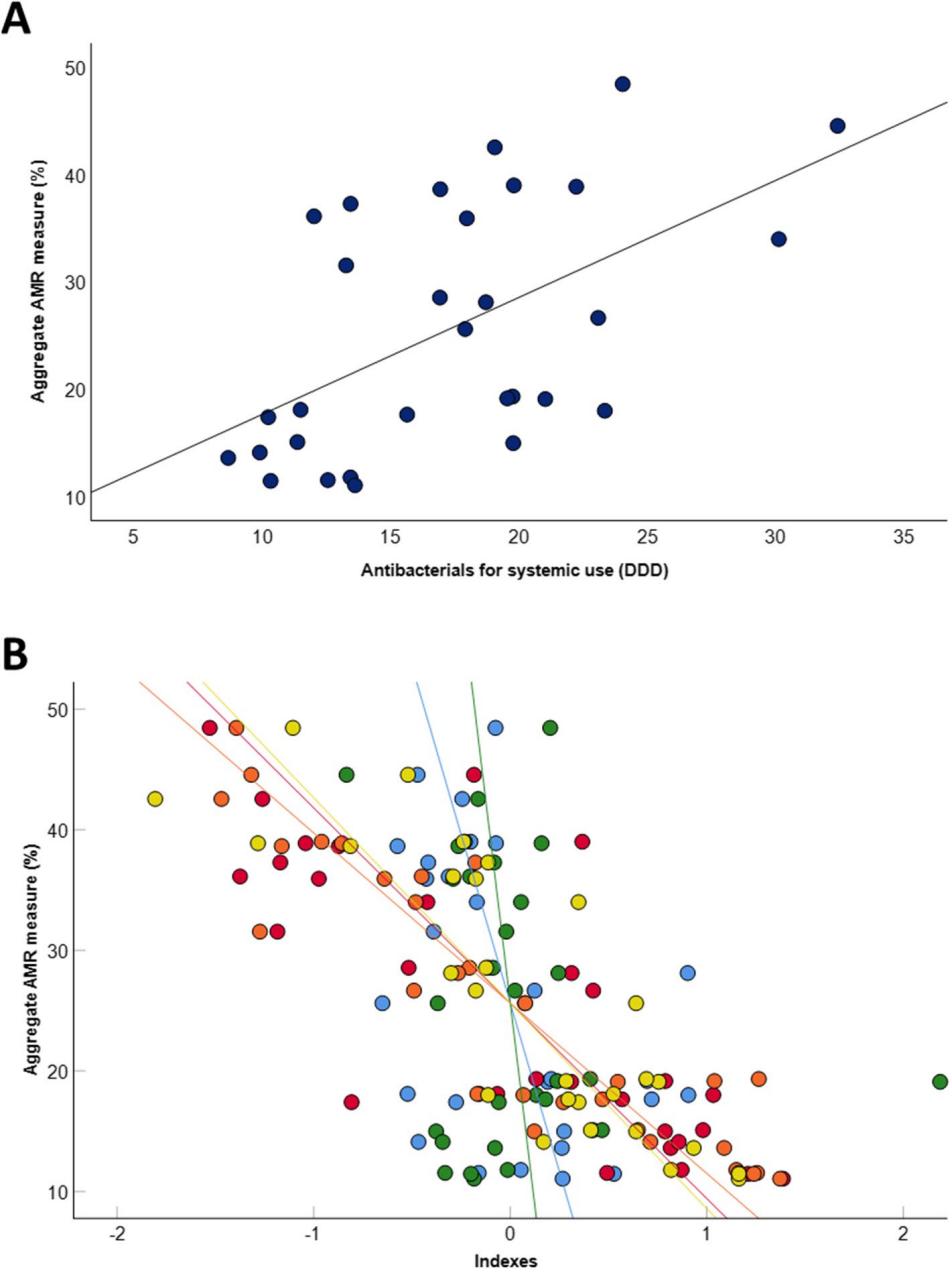
Relationships of AMR proportion with antibiotic use and indicators. **A** The scatter plot shows the relationship between the consumption of antibiotics for systemic use in the community (DDD per 1,000 residents and per day) and the aggregate measure of AMR. The line represents the linear regression line. **B** The superimposed scatter plot shows the relationships of demographic (in blue), health (in red), economic (in green), governance (in orange), and freedom (in yellow) indexes with the aggregate measure of AMR. The lines represent the linear regression lines

In further analyses, we evaluated the relationship of aggregate indexes and antibiotic consumption with the aggregate measure of AMR, which showed a median value of 22.5% (IQR = 21.3%) and some differences between European countries (Fig. 3). Taken on their own, the overall consumption of antibiotics in the community was positively related to the aggregate AMR measure (Fig. 4a), while indicators of health, governance, and freedom exhibited inverse relationships (Fig. 4b). In the multivariable regression model, an increase in health and governance indexes resulted in significant decrease of AMR proportion, also adjusting for the effect of antibiotic consumption. The increase of the latter, instead, was associated with increasing AMR proportions (Table 3).

**Table 3:** Multivariable linear regression of the relationship between aggregate indexes, antibiotic consumption and the aggregate measure of AMR

Indexes	Beta	Standard Error	*p*-value
Demographic	-0.065	0.324	0.843
Health	-0.632	0.185	0.002
Economic	0.086	0.288	0.767
Governance	-0.887	0.231	0.001
Freedom	0.057	0.039	0.154
Antibiotic use	0.329	0.128	0.017
R^2^	0.906		

**Fig. 5:**
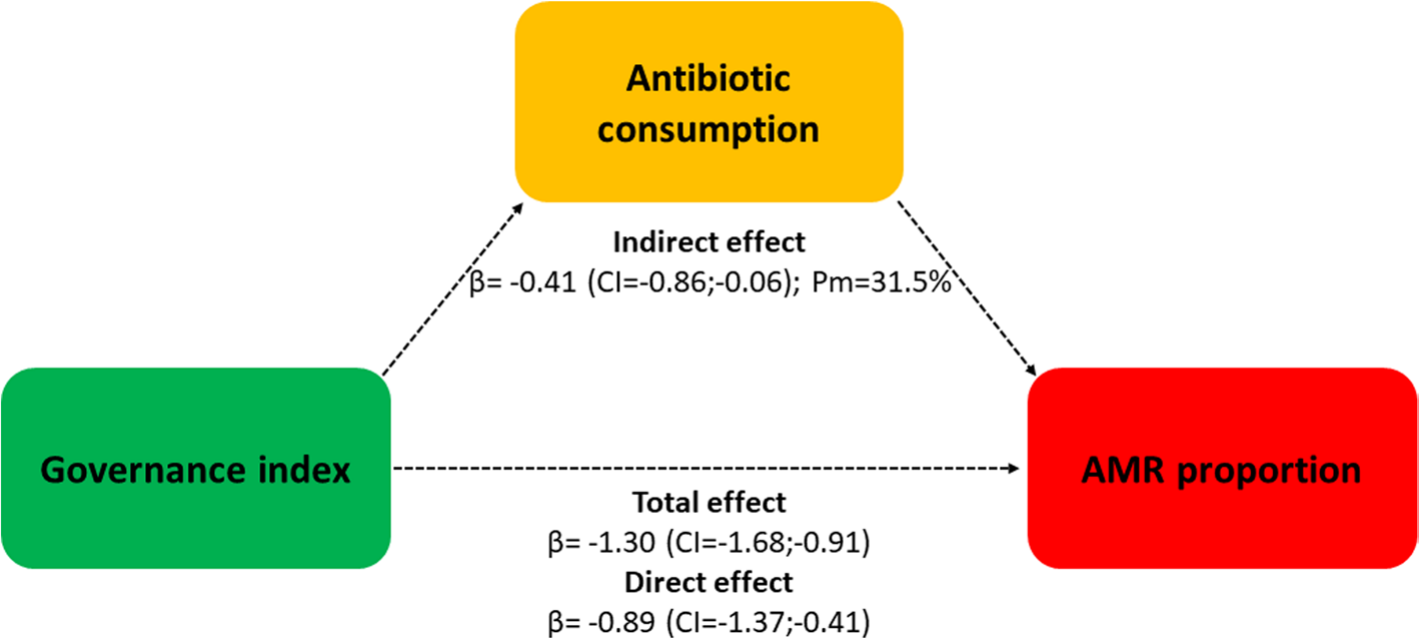
Analysis of the mediating effect of antibiotic consumption on the relationship between the governance index and AMR proportion. The percentage mediated (Pm) is expressed as the percentage of the total effect accounted for by the indirect effect

Overall, these findings pointed out a dual linear relationship of the governance index with antibiotic consumption and AMR. For this reason, we hypothesized that the overall consumption of antibiotics in the community might mediate the inverse relationship between the governance index and the aggregate measure of AMR. The mediation analysis, applied to test this hypothesis, confirmed that the governance index had an indirect effect on AMR via reducing antibiotic consumption. The total effect of the model was in fact significant (β = -1.30.; CI = -1.68;-0.91) and could be decomposed into a direct effect of the governance index on AMR (β = -0.89; CI = -1.37;-0.41) and an indirect effect through antibiotic consumption (β = -0.41; CI = -0.86;-0.06). These results suggested that antibiotic consumption partially mediated the relationship between the governance index and AMR, and that the mediator accounted for 31.5% of the total effect (Fig. 5).

### Differences between clusters of European countries

**Fig. 6:**
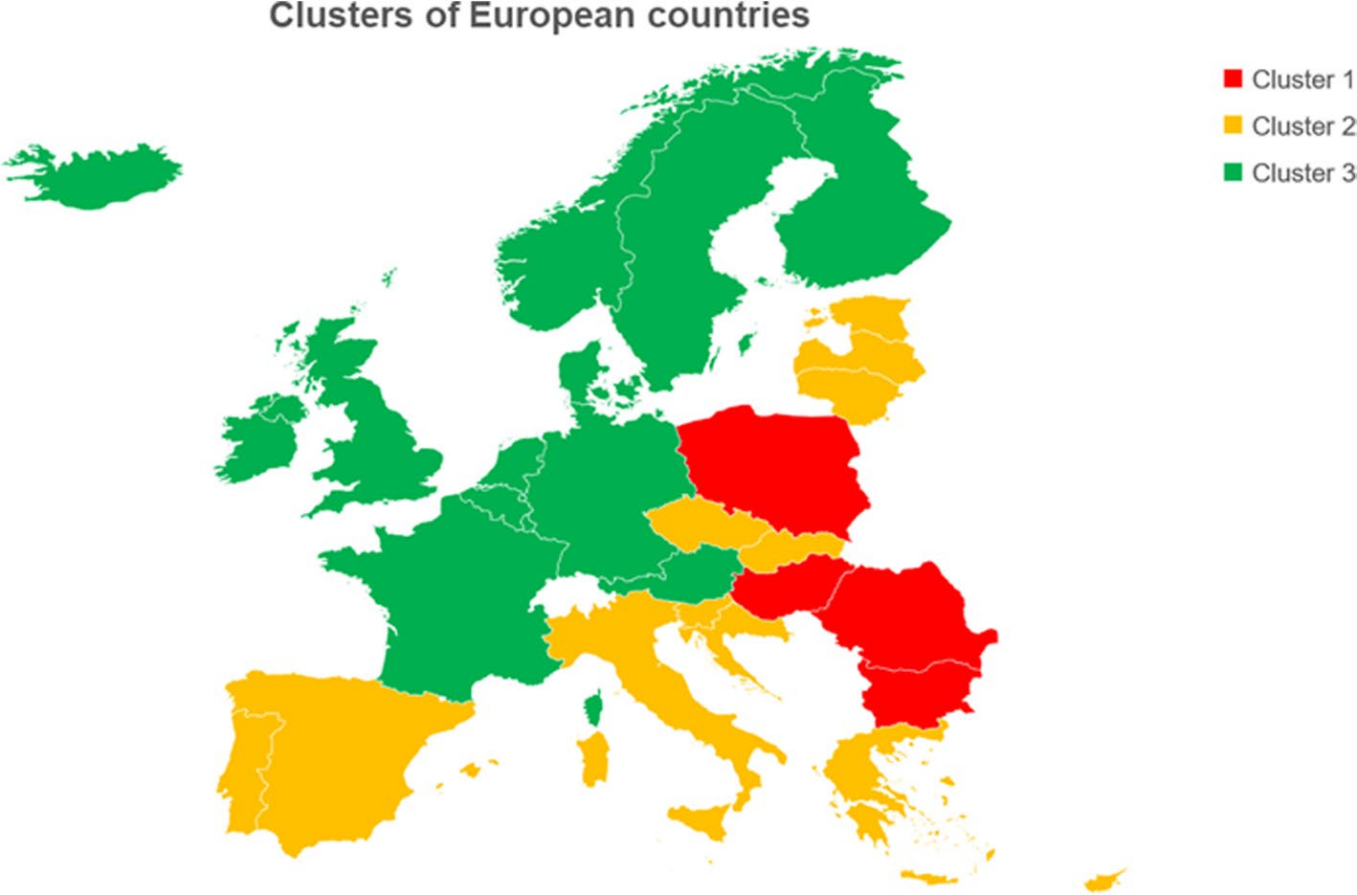
Clusters of European countries

**Fig. 7:**
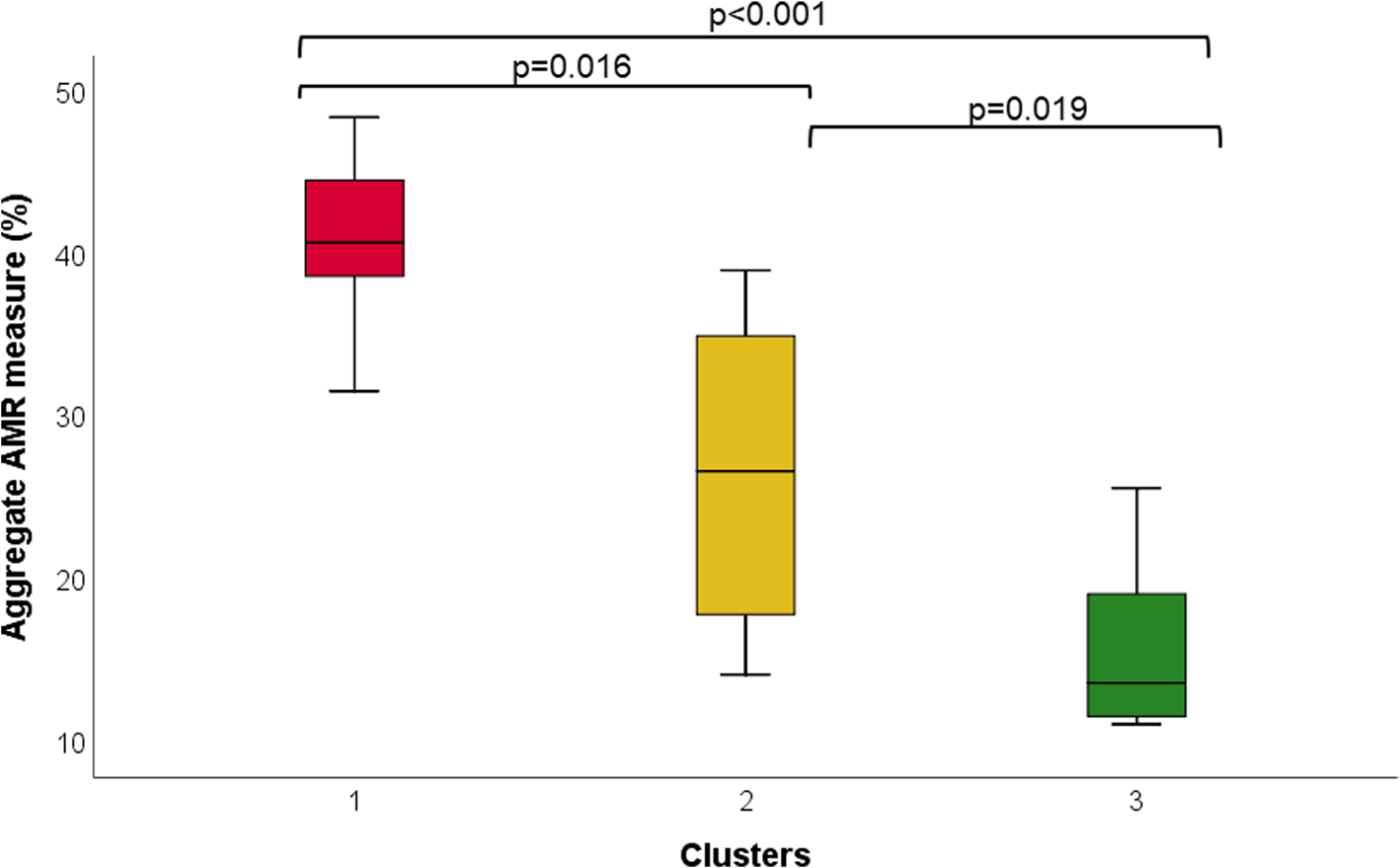
Comparison of AMR proportion between clusters. *P*-values based on the Kruskal–Wallis test

For the purpose of assessing the multivariate impact of socioeconomic, governance, and health factors on antibiotic consumption and AMR, we used the K-means clustering algorithm to divide Europe into different groups based on the above-mentioned indexes. This unsupervised method revealed three clusters of countries (Fig. 6) that mainly differed for demographic, health, governance, and freedom indexes (Table S6). Cluster 1 included Bulgaria, Hungary, Poland, and Romania, and exhibited the lowest values for the abovementioned indexes. Cluster 2 included 13 countries (Croatia, Cyprus, Czech Republic, Estonia, Greece, Italy, Latvia, Lithuania, Malta, Portugal, Slovak Republic, Slovenia, and Spain) characterized by medium indexes. Cluster 3 included the remaining 13 countries (Austria, Belgium, Denmark, Finland, France, Germany, Iceland, Ireland, Luxembourg, Netherlands, Norway, Sweden, United Kingdom), which reported the highest values for demographic, health, governance, and freedom indexes. The comparison between clusters revealed no significant difference in the consumption of antibiotics for systemic use in the community. However, the consumption of other beta-lactams (J01D) and quinolones (J01M) was the highest in cluster 1 and the lowest in cluster 3. The consumption of other antibiotics was instead the highest in cluster 3 (Table S7). As illustrated in Fig. 7, even the aggregate measure of AMR differed between clusters. In fact, cluster 1 (median = 40.7%; IQR = 8.7%) exhibited the highest aggregate value when compared with clusters 2 (median = 26.6%; IQR = 24.9%) and 3 (median = 13.6%; IQR = 14.5%).

## Discussion

In general, our analysis indicated that total and health expenditures, different aspects of quality governance, scores of political rights and civil liberties were all inversely related to the proportions of AMR across European countries. These findings add to the currently limited knowledge about the complex contribution of anthropological and socioeconomic factors to AMR. In particular, we observed that the governance was the most contributing factor to antibiotic consumption and AMR among all domains under investigation.

Several indicators of governance (i.e., voice and accountability, government effectiveness, regulatory quality, rule of law, and control of corruption) and freedom (i.e., political rights and civil liberties scores), in fact, showed similar inverse correlation with AMR levels in the bivariate analysis. Higher levels of governance indicators were also associated with lower antibiotic consumption. These findings were in agreement with those reported by previous studies at national and international levels [9, 13]. For example, it has been proposed how corruption is one of the main contributing factors to AMR [9, 21, 22].

Total GDP and health expenditure per capita were also inversely correlated with AMR proportions of different combinations between pathogens and antibiotics. However, the effect of country’s expenditures on AMR levels deserves further examination, because economic indicators may reflect other socioeconomic features. Our results from bivariate analysis, in fact, were partially in contrast with the global analysis carried out by Collignon and colleagues, which showed how high GDP per capita was associated with higher AMR levels [13]. Although this association was not significant in the multivariable analysis, the authors referred to the well-known association between high GDP per capita and high antibiotic consumption in support of their claim [23]. Our analysis of European countries, however, did not find any correlation between GPD per capita and antibiotic consumption. For this reason, our hypothesis was that more directly relevant variables, such as health expenditure, may explain the beneficial effects of GDP on AMR levels observed in our study. The response to AMR, in fact, would benefit from higher health expenditure. In this context, it is also worth mentioning that earlier studies of European data showed that levels of AMR rose with increasing private health spending [9, 13]. However, this indicator could conceivably reflect the business volume of the private health sector, provided by individual health professionals and healthcare companies. According to previous data [24], there may be differences between the public and private health sectors in terms of regulation and antibiotic consumption; differences that would help explain the observed relationship between private health spending and AMR levels.

Overall, our analysis confirms AMR as a multifaced issue that requires concerted and informed approaches. To disentangle the multifactorial contribution to antibiotic use and AMR, we developed five indexes and an aggregate measure of AMR. Multivariable analysis showed that higher indexes of governance and health were significantly associated with lower aggregate levels of AMR. This was true even when considering the detrimental effect of antibiotic consumption on AMR. Among all indexes, the one related to governance was also associated with lower antibiotic consumption in the community. Given the dual action of the governance index, we evaluated the mediating effect of antibiotic consumption on AMR levels. Mediation analysis is increasingly being applied in many fields of research, including epidemiological research [18], and in our case can be used to investigate the antibiotic consumption as a mediator of the relationship between governance and AMR. Surprisingly, the mediation analysis estimated that only a third of the reduction in AMR can be attributed to the decrease in antibiotic consumption associated with increased governance index. This means that there may be other factors other than antibiotic use volumes that explain how governance quality affects AMR [7]. It is undeniable that antibiotic use is one of the main contributing factors to AMR, as also proven by our analysis. Nevertheless, uncontrolled spread of resistant pathogens, defined by Collignon and colleagues as "contagion" [7], might also be significant. In fact, one could expect higher levels of AMR in countries with conditions that favor the spread of resistant pathogens in the healthcare setting and in the community. A number of factors could contribute to this problem, including low hygiene awareness, weak governance systems, and poor sanitation management in all sectors (e.g., health facilities, houses, water treatment plants, and the food supply chain). This hypothesis is also supported by the observation of high AMR levels in countries with low antibiotic consumption, but weak governments, low incomes, and poor infrastructures [9]. In addition, the inefficiency of governance may contribute to a lack of monitoring of the appropriateness of antibiotic prescriptions [21, 22].

With so many factors contributing to AMR, conventional methods of data analysis are often inadequate to address the issue. Hence, we grouped countries by socioeconomics, governance, and health characteristics using a multivariate approach. When we combined countries into different clusters, antibiotic consumption and AMR levels were the highest in countries with the lowest health, governance, and freedom indexes (i.e., Bulgaria, Hungary, Poland, and Romania). This evidence increased our confidence in the robustness of the findings.

Our analysis had several limitations to be discussed. First, it was limited to European countries and data for one year. Although a broader approach could provide meaningful evidence at global level, there was the need for using data that were almost complete and consistent between countries. Just considering one year, in our analysis there were some countries with incomplete data on AMR, especially for some combinations between pathogen and antibiotic (e.g., *Acinetobacter* spp. resistance to fluoroquinolones, aminoglycosides, carbapenems). Missing data were imputed with the average values obtained from countries with available data. Although this was considered the best way to solve the issue, we were unable to identify and manage factors that determined the presence of missing values. The hypothesis that countries reporting data have the best surveillance systems suggests that imputing missing values might have introduced a confounding effect. However, the reasons behind the presence of missing values can be various and not always related to the effectiveness of surveillance systems or to the development of the country. For instance, countries with missing data for *Acinetobacter* spp. were Estonia, Iceland, Luxembourg, and Malta, which are heterogeneous in terms of socio-demographic and organizational features. In the near future, it would be interesting to replicate our analyses taking into account other factors determining the effectiveness of surveillance systems and/or comparing countries from other regions of the world. Additionally, even when complete, AMR data might be underestimated or overestimated without representative surveillance systems. The same applies, presumably, to data on antibiotic consumption, which might be underestimated due to the volume of antibiotics purchased through other sales channels. For the same reasons, Collignon and colleagues' analysis [13] covered just over seventy countries on a global scale and aggregated data from different years and sources to obtain a representative and almost complete dataset.

Second, we summarized the overall set of indicators using aggregate indexes that captured domains that may contribute to antibiotic consumption and AMR. The choice of creating ad-hoc aggregate indexes was motivated by the presence of many possible contributing indicators, which were also interrelated to each other. It was not our intention, in fact, to provide evidence on specific indicators, but rather to evaluate what are the main domains associated with antibiotic consumption and AMR. Moreover, the methodology behind the creation of aggregate indexes from available indicators was borrowed from Collignon and colleagues [13]. There is no doubt that other composite indicators could be used to summarize sociodemographic and economic characteristics of European countries (e.g., the Socio-demographic Index), however, we decided to create ad-hoc indexes reflecting the specific demographic, health, economic, governance, and freedom domains. Third, additional factors that might contribute to antibiotic use and AMR were not considered. For instance, we could not use data on infrastructures, education, healthcare access, sanitation, and public awareness on antibiotic consumption and AMR. Further, we did not take into account data on antibiotic consumption in hospitals and in the animal sector, which account for the largest part of total antibiotic consumption, nor we did examine antibiotic stewardship and infection prevention protocols. For instance, a country might report low antibiotic consumption in the human sector – whether in hospital or in the community – but high consumption in the animal sector. All these factors not considered in our analyses, or at least some of them, might explain the remaining part of the effect of governance on AMR. For this reason, future studies should be encouraged to approach the problem from a One Health perspective.

## Conclusions

Despite these limitations, the present study was the first applying bivariate, multivariable, and multivariate analyses to study how socio-economic, governance, and health indicators shape AMR levels across European countries. Searching PubMed for articles published from the inception to November 2022, we identified only the study by Collignon and colleagues that applied a multivariable analysis to the above factors contributing to AMR [13]. However, none of the retrieved articles examined the possible mediating effect of antibiotic consumption, nor did it apply a multivariate analysis. Originally inspired by this research, our analysis was designed to provide a better understanding of factors contributing to antibiotic use and AMR in Europe. Among all domains under consideration, the governance of a country was associated with both antibiotic consumption and AMR levels, suggesting a potential mediating effect. However, decreasing antibiotic consumption only accounted for a part of the total effect of better governance on reduced AMR proportions. These findings could be – at least partially – explained by the contagion theory, for which other factors or conditions sustain the uncontrolled spread of resistant pathogens in countries with poor governance. As a result of this evidence, reducing antibiotic use alone is unlikely to solve the AMR problem, and more interventions are needed to increase governance efficiency at global level.

## Supplementary Information

**Additional file 1:** **Table S1.** Descriptive statistics of antibiotic consumption. **Table S2.** Spearman correlation coefficients between indicators and antibiotic consumptions. **Table S3.** AMR proportions for combinations between pathogens and antibiotics. **Table S4.** Spearman correlation coefficients between indicators and proportion of AMR for each combination between pathogen and antibiotic. **Table S5.** Spearman correlation coefficients between aggregate indexes and antibiotic consumption. **Table S6.** Differences in indexes between clusters. **Table S7.** Differences in antibiotic use between clusters. **Figure S1.** Demographic index across European countries. **Figure S2.** Health index across European countries. **Figure S3.** Economic index across European countries. **Figure S4.** Governance index across European countries. **Figure S5.** Freedom and rights index across European countries.

## Data Availability

The datasets used and/or analysed during the current study are available from the corresponding author on reasonable request.

## Abbreviations

AMR: Antimicrobial resistance
LMICs: Low- and middle-income countries
HICs: High-income countries
EU/EEA: European Union/European economic area
GDP: Gross domestic product
ECDC: European centre for disease prevention and control
DDD: Defined daily dose
SD: Standard deviation
SE: Standard error
CI: Confidence interval
IQR: Interquartile range
