# Supplementary material for: Socio-economic, governance and health indicators shaping antimicrobial resistance: an ecological analysis of 30 european countries
Source: Global Health. 2023 Feb 24;19:12. doi: 10.1186/s12992-023-00913-0 (PMC9951828; doi:10.1186/s12992-023-00913-0)

**Supplementary Materials**

**Table of Contents**

**Table S1.** Descriptive statistics of antibiotic consumption.

**Table S2.** Spearman correlation coefficients between indicators and antibiotic consumptions.

**Table S3.** AMR proportions for combinations between pathogens and antibiotics.

**Table S4.** Spearman correlation coefficients between indicators and proportion of AMR for each combination between pathogen and antibiotic.

**Table S5.** Spearman correlation coefficients between aggregate indexes and antibiotic consumption.

**Table S6.** Differences in indexes between clusters.

**Table S7.** Differences in antibiotic use between clusters.

**Figure S1.** Demographic index across European countries.

**Figure S2.** Health index across European countries.

**Figure S3.** Economic index across European countries.

**Figure S4.** Governance index across European countries.

**Figure S5.** Freedom and rights index across European countries.

**Table S1. Descriptive statistics of antibiotic consumption.**

| **Antibiotic class** | **Countries with available data** | **Mean** | **Standard deviation** | **Minimum** | **Maximum** | **Median** | **IQR** |
| --- | --- | --- | --- | --- | --- | --- | --- |
| **J01** | 30 | 17.3 | 5.8 | 8.7 | 32.4 | 17.4 | 7.7 |
| **J01C** | 30 | 7.4 | 2.8 | 2.8 | 13.3 | 6.7 | 4.2 |
| **J01D** | 30 | 2.2 | 1.9 | 0.03 | 7.6 | 1.9 | 2.2 |
| **J01G** | 30 | 0.03 | 0.03 | 0.00 | 0.14 | 0.02 | 0.02 |
| **J01M** | 30 | 1.4 | 1.2 | 0.3 | 5.8 | 1.1 | 1.3 |
| **J01X** | 30 | 1.1 | 0.9 | 0.01 | 4.3 | 0.9 | 0.8 |

**Table S2. Spearman correlation coefficients between indicators and antibiotic consumptions.**

| **Indicators** | **J01** | **J01C** | **J01D** | **J01G** | **J01M** | **J01X** |
| --- | --- | --- | --- | --- | --- | --- |
| **D1** | 0.15 | 0.15 | 0.03 | 0.19 | 0.03 | -0.08 |
| **D2** | 0.14 | 0.07 | -0.04 | -0.02 | -0.10 | -0.03 |
| **D3** | 0.13 | 0.13 | 0.15 | 0.28 | 0.16 | 0.01 |
| **D4** | 0.06 | 0.12 | -0.17 | 0.06 | -0.06 | 0.11 |
| **D5** | 0.10 | 0.00 | -0.22 | 0.08 | -0.42 | 0.41 |
| **D6** | -0.06 | -0.09 | -0.40 | -0.07 | -0.38 | 0.31 |
| **D7** | -0.18 | -0.06 | -0.48 | 0.13 | -0.48 | -0.13 |
| **H1** | -0.27 | 0.07 | -0.43 | -0.09 | -0.45 | 0.08 |
| **H2** | -0.19 | 0.10 | -0.54 | -0.23 | -0.58 | 0.42 |
| **H3** | 0.14 | 0.35 | -0.29 | -0.12 | -0.19 | 0.29 |
| **E1** | 0.00 | 0.12 | -0.22 | 0.04 | -0.28 | 0.19 |
| **E2** | -0.19 | 0.07 | -0.54 | -0.19 | -0.59 | 0.47 |
| **E3** | 0.16 | -0.02 | 0.24 | 0.14 | 0.24 | -0.13 |
| **E4** | 0.16 | -0.04 | 0.23 | 0.12 | 0.28 | -0.25 |
| **G1** | -0.33 | -0.08 | -0.60 | -0.32 | **-0.69** | 0.54 |
| **G2** | -0.40 | -0.22 | -0.30 | -0.34 | -0.39 | 0.35 |
| **G3** | -0.53 | -0.25 | **-0.70** | -0.34 | **-0.76** | 0.43 |
| **G4** | -0.50 | -0.35 | -0.63 | -0.24 | **-0.79** | 0.52 |
| **G5** | -0.53 | -0.21 | -0.67 | -0.33 | **-0.77** | 0.45 |
| **G6** | -0.43 | -0.20 | -0.63 | -0.34 | **-0.75** | 0.51 |
| **F1** | -0.39 | -0.15 | **-0.69** | -0.36 | **-0.70** | 0.46 |
| **F2** | -0.32 | -0.09 | -0.54 | -0.34 | -0.59 | 0.58 |

Significant results after Bonferroni correction (p-values < 3.788 x 10^-4^) are indicated in bold font.

**Table S3. AMR proportions for combinations between pathogens and antibiotics.**

| **Combination of pathogen and antibiotic** | **Countries with available data** | **Mean** | **Standard deviation** | **Minimum** | **Maximum** | **Median** | **IQR** |
| --- | --- | --- | --- | --- | --- | --- | --- |
| **Acinetobacter spp. resistant to fluoroquinolones** | 26 | 43.4 | 37.1 | 0.0 | 95.8 | 32.6 | 74.7 |
| **Acinetobacter spp. resistant to aminoglycosides** | 26 | 38.8 | 34.6 | 0.0 | 92.1 | 33.7 | 70.1 |
| **Acinetobacter spp. resistant to carbapenems** | 26 | 39.5 | 37.1 | 0.0 | 92.3 | 31.1 | 73.8 |
| **Acinetobacter spp. combined resistance** | 26 | 35.1 | 34.4 | 0.0 | 91.4 | 29.5 | 65.3 |
| **K. pneumoniae resistant to carbapenems** | 29 | 7.6 | 13.3 | 0.0 | 58.3 | 1.1 | 8.8 |
| **K. pneumoniae resistance to fluoroquinolones** | 30 | 32.1 | 20.2 | 4.3 | 66.9 | 36.7 | 39.3 |
| **K. pneumoniae resistant to 3^rd^ generation cephalosporins** | 30 | 32.3 | 22.1 | 4.3 | 75.7 | 36.7 | 41.9 |
| **K. pneumoniae resistant to aminoglycosides** | 30 | 23.9 | 18.0 | 3.5 | 57.3 | 28.3 | 37.4 |
| **K. pneumoniae combined resistance** | 30 | 20.3 | 17.1 | 0.0 | 53.1 | 25.3 | 33.7 |
| **E. coli resistant to aminopenicillins** | 29 | 55.9 | 8.6 | 35.5 | 71.7 | 57.8 | 11.2 |
| **E. coli resistant to fluoroquinolones** | 30 | 23.7 | 9.3 | 11.3 | 43.5 | 23.1 | 12.2 |
| **E. coli resistant to 3^rd^ generation cephalosporins** | 30 | 14.8 | 7.3 | 6.2 | 38.6 | 14.1 | 10.4 |
| **MRSA** | 30 | 14.9 | 12.9 | 1.0 | 46.9 | 12.5 | 17.4 |
| **P. aeruginosa resistant to piperacillin and tazobactam** | 29 | 17.4 | 11.7 | 2.3 | 52.8 | 16.7 | 14.0 |
| **P. aeruginosa resistant to fluoroquinolones** | 30 | 19.1 | 13.1 | 4.5 | 52.2 | 18.9 | 17.8 |
| **P. aeruginosa resistant to carbapenems** | 30 | 17.6 | 13.4 | 0.0 | 55.4 | 16.3 | 14.0 |
| **P. aeruginosa resistant to ceftazidime** | 30 | 15.9 | 11.8 | 3.5 | 52.2 | 16.0 | 12.7 |
| **S. pneumoniae resistant to penicillins** | 28 | 3.3 | 5.0 | 0.0 | 19.8 | 1.6 | 3.7 |
| **S. pneumoniae resistant to macrolides** | 28 | 13.6 | 7.9 | 3.5 | 30.4 | 12.4 | 11.4 |
| **E. faecium resistant to aminopenicillins** | 28 | 89.5 | 6.0 | 75.1 | 100.0 | 90.2 | 9.4 |
| **E. faecium resistant to vancomycin** | 28 | 18.7 | 17.2 | 0.0 | 50.0 | 19.8 | 33.7 |
| **E. faecium resistant to high level gentamicin** | 27 | 41.6 | 22.9 | 0.0 | 89.3 | 50.9 | 35.0 |
| **E. faecalis resistant to aminopenicillins** | 30 | 1.3 | 2.2 | 0.0 | 9.8 | 0.4 | 1.2 |
| **E. faecalis resistant to vancomycin** | 30 | 1.0 | 1.8 | 0.0 | 8.1 | 0.3 | 1.3 |
| **E. faecalis resistant to high level gentamicin** | 28 | 23.2 | 12.5 | 0.0 | 44.1 | 24.0 | 18.0 |

**Table S4. Spearman correlation coefficients between indicators and proportion of AMR for each combination between pathogen and antibiotic.**

| **Pathogen** | **Antibiotic class** | **D1** | **D2** | **D3** | **D4** | **D5** | **D6** | **D7** | **H1** | **H2** | **H3** | **E1** | **E2** | **E3** | **E4** | **G1** | **G2** | **G3** | **G4** | **G5** | **G6** | **F1** | **F2** |
| --- | --- | --- | --- | --- | --- | --- | --- | --- | --- | --- | --- | --- | --- | --- | --- | --- | --- | --- | --- | --- | --- | --- | --- |
| **Acinetobacter spp.** | **Fluoroquinolones** | -0.17 | -0.20 | -0.15 | -0.10 | -0.38 | -0.56 | -0.21 | **-0.74** | **-0.84** | -0.46 | -0.55 | **-0.83** | 0.38 | 0.55 | **-0.84** | -0.51 | **-0.86** | **-0.84** | **-0.89** | **-0.87** | **-0.76** | **-0.76** |
|  | **Aminoglycosides** | -0.14 | -0.07 | -0.18 | -0.08 | -0.40 | -0.55 | -0.27 | -0.66 | **-0.81** | -0.43 | -0.51 | **-0.82** | 0.30 | 0.43 | **-0.82** | -0.49 | **-0.84** | **-0.84** | **-0.87** | **-0.86** | **-0.76** | **-0.76** |
|  | **Carbapenems** | -0.14 | -0.07 | -0.21 | -0.08 | -0.44 | -0.60 | -0.25 | **-0.70** | **-0.82** | -0.44 | -0.52 | **-0.82** | 0.33 | 0.50 | **-0.82** | -0.50 | **-0.82** | **-0.83** | **-0.87** | **-0.86** | **-0.74** | **-0.75** |
|  | **Combined** | -0.09 | -0.04 | -0.16 | -0.12 | -0.46 | -0.55 | -0.28 | -0.67 | **-0.82** | -0.43 | -0.49 | **-0.83** | 0.30 | 0.46 | **-0.82** | -0.50 | **-0.84** | **-0.84** | **-0.88** | **-0.86** | **-0.76** | **-0.77** |
| **Klebsiella pneumoniae** | **Carbapenems** | 0.17 | 0.06 | 0.17 | -0.13 | -0.33 | -0.33 | -0.45 | -0.24 | -0.43 | -0.09 | -0.09 | -0.47 | 0.09 | 0.18 | -0.53 | -0.33 | **-0.74** | **-0.76** | **-0.72** | -0.64 | -0.59 | -0.52 |
|  | **Fluoroquinolones** | 0.13 | -0.06 | 0.12 | -0.29 | -0.45 | -0.53 | -0.22 | -0.59 | **-0.80** | -0.48 | -0.22 | **-0.82** | 0.23 | 0.50 | **-0.85** | -0.45 | **-0.90** | **-0.84** | **-0.90** | **-0.87** | **-0.74** | **-0.77** |
|  | **Third generation cephalosporins** | 0.16 | -0.02 | 0.14 | -0.25 | -0.42 | -0.50 | -0.23 | -0.56 | **-0.78** | -0.46 | -0.18 | **-0.80** | 0.22 | 0.48 | **-0.83** | -0.49 | **-0.90** | **-0.83** | **-0.91** | **-0.85** | **-0.72** | **-0.76** |
|  | **Aminoglycosides** | 0.08 | 0.00 | 0.03 | -0.23 | -0.38 | -0.45 | -0.14 | -0.61 | **-0.77** | -0.49 | -0.28 | **-0.80** | 0.28 | 0.51 | **-0.86** | -0.40 | **-0.89** | **-0.83** | **-0.89** | **-0.87** | **-0.77** | **-0.76** |
|  | **Combined** | 0.15 | -0.01 | 0.08 | -0.33 | -0.42 | -0.50 | -0.18 | -0.60 | **-0.82** | -0.51 | -0.22 | **-0.84** | 0.21 | 0.49 | **-0.85** | -0.47 | **-0.89** | **-0.81** | **-0.89** | **-0.85** | **-0.70** | **-0.76** |
| **Escherichia coli** | **Aminopenicillins** | 0.05 | -0.02 | 0.19 | 0.18 | -0.06 | -0.27 | -0.26 | -0.44 | -0.44 | -0.14 | -0.12 | -0.41 | 0.44 | 0.46 | -0.55 | -0.33 | -0.66 | -0.60 | **-0.70** | -0.61 | -0.55 | -0.48 |
|  | **Fluoroquinolones** | 0.01 | -0.18 | 0.27 | -0.03 | -0.30 | -0.42 | -0.50 | -0.51 | -0.64 | -0.28 | -0.26 | -0.64 | 0.36 | 0.41 | **-0.76** | **-0.41** | **-0.86** | **-0.81** | **-0.88** | **-0.83** | **-0.75** | -0.61 |
|  | **Third generation cephalosporins** | 0.01 | -0.13 | 0.13 | -0.17 | -0.37 | -0.50 | -0.36 | -0.62 | **-0.76** | -0.49 | -0.30 | **-0.75** | 0.34 | 0.49 | **-0.82** | -0.45 | **-0.86** | **-0.80** | **-0.90** | **-0.85** | **-0.74** | **-0.69** |
| **Staphylococcus aureus** | **Methicillin** | 0.12 | -0.02 | 0.15 | -0.20 | -0.39 | -0.52 | -0.33 | -0.45 | -0.65 | -0.28 | -0.21 | **-0.67** | 0.28 | 0.45 | **-0.75** | -0.44 | **-0.85** | **-0.87** | **-0.87** | **-0.85** | **-0.66** | -0.64 |
| **Pseudomonas aeruginosa** | **Piperacillin and Tazobactam** | 0.10 | 0.08 | -0.11 | -0.27 | -0.45 | -0.55 | -0.02 | -0.49 | **-0.80** | -0.52 | -0.30 | **-0.83** | 0.27 | 0.56 | **-0.85** | -0.52 | **-0.83** | **-0.80** | **-0.85** | **-0.84** | **-0.71** | **-0.75** |
|  | **Fluoroquinolones** | 0.32 | 0.07 | 0.09 | -0.36 | -0.57 | -0.58 | -0.14 | -0.43 | **-0.79** | -0.50 | -0.05 | **-0.83** | 0.10 | 0.45 | **-0.84** | -0.51 | **-0.85** | **-0.79** | **-0.83** | **-0.82** | **-0.66** | **-0.75** |
|  | **Carbapenems** | 0.15 | 0.09 | -0.10 | -0.29 | -0.49 | -0.65 | -0.23 | -0.49 | **-0.78** | -0.53 | -0.18 | **-0.83** | 0.14 | 0.43 | **-0.83** | -0.51 | **-0.79** | **-0.78** | **-0.79** | **-0.80** | -0.63 | **-0.75** |
|  | **Ceftazidime** | 0.05 | 0.05 | -0.12 | -0.23 | -0.43 | -0.60 | -0.03 | -0.52 | **-0.79** | -0.54 | -0.33 | **-0.83** | 0.29 | 0.56 | **-0.90** | -0.44 | **-0.86** | **-0.86** | **-0.86** | **-0.89** | **-0.75** | **-0.77** |
| **Streptococcus pneumoniae** | **Penicillins** | 0.17 | 0.16 | 0.17 | -0.08 | -0.31 | -0.07 | -0.46 | -0.25 | -0.33 | -0.04 | 0.01 | -0.34 | 0.06 | 0.05 | -0.34 | -0.29 | -0.35 | -0.37 | -0.41 | -0.37 | -0.47 | -0.36 |
|  | **Macrolides** | 0.11 | 0.18 | 0.02 | -0.02 | -0.26 | -0.21 | -0.30 | -0.17 | -0.41 | 0.01 | -0.08 | -0.44 | 0.26 | 0.34 | -0.53 | -0.25 | **-0.70** | **-0.69** | -0.64 | -0.63 | **-0.68** | -0.48 |
| **Enterococcus faecium** | **Aminopenicillins** | -0.18 | -0.14 | -0.19 | -0.21 | -0.27 | -0.31 | 0.06 | -0.44 | -0.58 | -0.62 | -0.34 | -0.52 | 0.40 | 0.62 | -0.55 | -0.23 | -0.41 | -0.33 | -0.49 | -0.48 | -0.44 | -0.55 |
|  | **Vancomycin** | -0.13 | -0.12 | -0.04 | -0.15 | -0.10 | -0.58 | -0.07 | -0.59 | -0.57 | -0.55 | -0.36 | -0.55 | 0.45 | 0.54 | -0.63 | -0.31 | -0.57 | -0.54 | -0.64 | -0.65 | -0.52 | -0.59 |
|  | **High level gentamicin** | 0.22 | 0.18 | -0.04 | -0.12 | -0.09 | -0.06 | 0.35 | -0.25 | -0.36 | -0.37 | 0.13 | -0.30 | 0.19 | 0.38 | -0.34 | -0.08 | -0.27 | -0.12 | -0.27 | -0.31 | -0.29 | -0.41 |
| **Enterococcus faecalis** | **Aminopenicillins** | -0.11 | -0.08 | -0.05 | -0.04 | -0.16 | 0.02 | 0.07 | -0.26 | -0.13 | -0.23 | -0.12 | -0.13 | 0.02 | 0.04 | -0.18 | -0.01 | -0.02 | 0.00 | -0.05 | -0.04 | -0.29 | -0.22 |
|  | **Vancomycin** | 0.27 | 0.00 | 0.17 | -0.20 | -0.13 | -0.22 | 0.02 | -0.21 | -0.33 | -0.28 | 0.15 | -0.32 | -0.05 | 0.26 | -0.29 | -0.48 | -0.30 | -0.24 | -0.36 | -0.29 | -0.07 | -0.34 |
|  | **High level gentamicin** | 0.19 | 0.16 | -0.11 | -0.20 | -0.45 | -0.47 | -0.02 | -0.42 | -0.66 | -0.49 | 0.00 | -0.64 | 0.30 | 0.60 | -0.67 | -0.28 | -0.56 | -0.47 | -0.56 | -0.59 | -0.60 | -0.64 |

Significant results after Bonferroni correction (p-values < 9.091 x 10^-5^) are indicated in bold font.

**Table S5. Spearman correlation coefficients between aggregate indexes and antibiotic consumption**.

| **Indexes** | **J01** | **J01C** | **J01D** | **J01G** | **J01M** | **J01X** |
| --- | --- | --- | --- | --- | --- | --- |
| **Demographic** | 0.12 | 0.04 | -0.34 | 0.11 | -0.42 | 0.22 |
| **Health** | -0.21 | 0.10 | -0.52 | -0.18 | **-0.55** | 0.30 |
| **Economic** | 0.20 | 0.06 | -0.05 | 0.14 | -0.06 | -0.07 |
| **Governance** | **-0.69** | -0.22 | **-0.65** | -0.36 | **-0.76** | 0.54 |
| **Freedom** | -.38 | -0.13 | **-0.64** | -0.37 | **-0.68** | 0.54 |

Significant results after Bonferroni correction (p-values < 1.667 x 10^-3^) are indicated in bold font

**Table S6. Differences in indexes between clusters.** Results are reported as median (interquartile range).

| **Indexes** | **Cluster 1** | **Cluster 2** | **Cluster 3** | **p-value** |
| --- | --- | --- | --- | --- |
| **Demographic** | -0.16 (0.28) | -0.31 (0.36) | 0.27 (0.49) | 0.002 |
| **Health** | -1.22 (0.39) | -0.42 (1.12) | 0.82 (0.48) | <0.001 |
| **Economic** | 0.07 (0.32) | -0.16 (0.26) | -0.01 (0.59) | 0.218 |
| **Governance** | -1.33 (0.49) | -0.45 (0.63) | 1.04 (0.74) | <0.001 |
| **Freedom** | -1.55 (1.76) | -0.18 (0.64) | 0.69 (0.76) | <0.001 |

**Table S7. Differences in antibiotic use and clusters.** Results are reported as median (interquartile range).

| **Antibiotic class** | **Antibiotic consumption (DDD per 1,000 residents per day)** | | | **p-value** |
| --- | --- | --- | --- | --- |
|  | **Cluster 1** | **Cluster 2** | **Cluster 3** |  |
| **J01** | 20.6 (10.1) | 16.9 (8.3) | 13.6 (8.3) | 0.212 |
| **J01C** | 7.2 (5.9) | 6.7 (4.5) | 8.3 (4.6) | 0.935 |
| **J01D** | 3.9 (2.9) | 2.3 (2.2) | 1.2 (1.7) | 0.005 |
| **J01G** | 0.04 (0.07) | 0.02 (0.02) | 0.01 (0.01) | 0.139 |
| **J01M** | 2.4 (1.7) | 1.0 (1.2) | 0.6 (1.6) | 0.005 |
| **J01X** | 0.6 (1.6) | 0.7 (0.6) | 1.4 (0.7) | 0.003 |

**Supplementary Figure 1. Demographic index across European countries.** Map shows levels of the demographic index from low (in red) to high (in green). The index was calculated as the average of standardized demographic indicators (total population; land area; population density; annual population growth; population aged > 40; urban population; fertility rate).


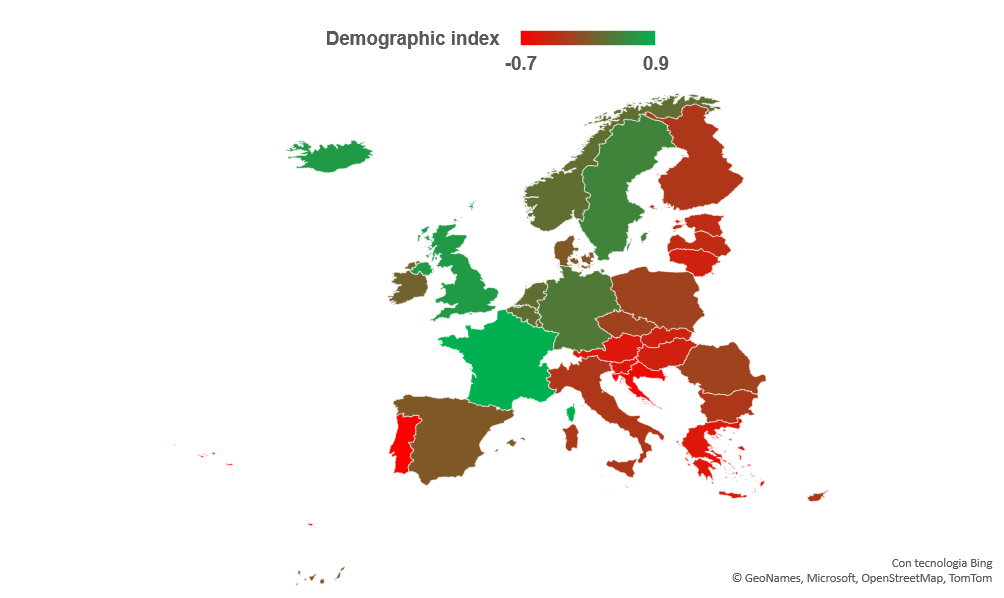


**Supplementary Figure 2. Health index across European countries.** Map shows levels of the health index from low (in red) to high (in green). The index was calculated as the average of standardized health indicators (health expenditure; health expenditure per capita; life expectancy at birth).


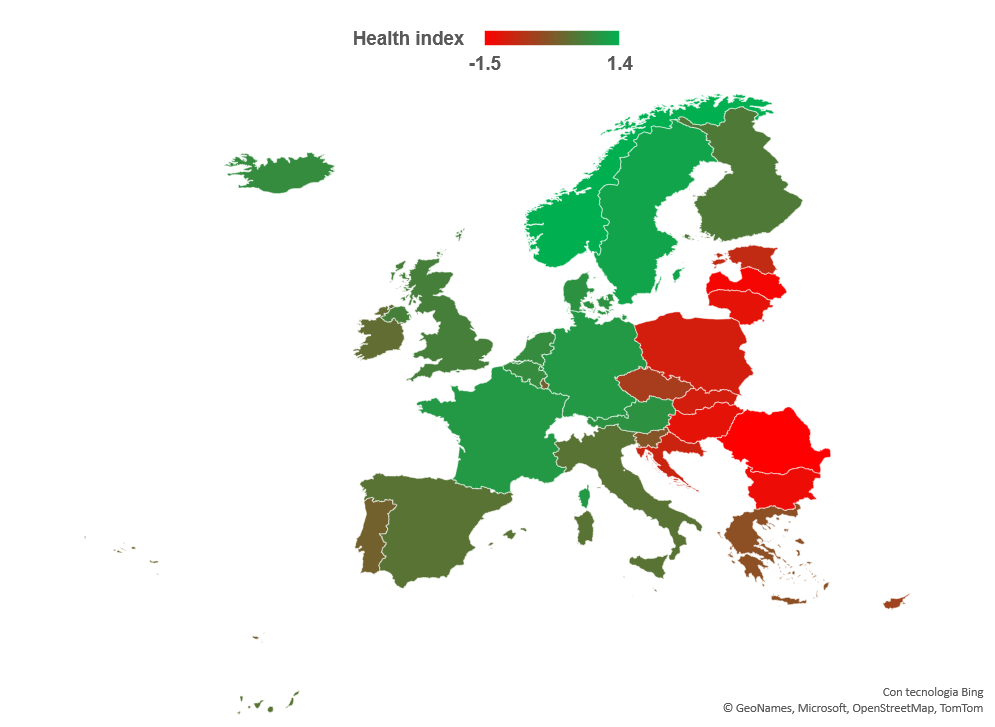


**Supplementary Figure 3. Economic index across European countries.** Map shows levels of the economic index from low (in red) to high (in green). The index was calculated as the average of standardized economic indicators (GDP; GDP per capita; 5-year average GDP growth; 5-year average GDP growth per capita).


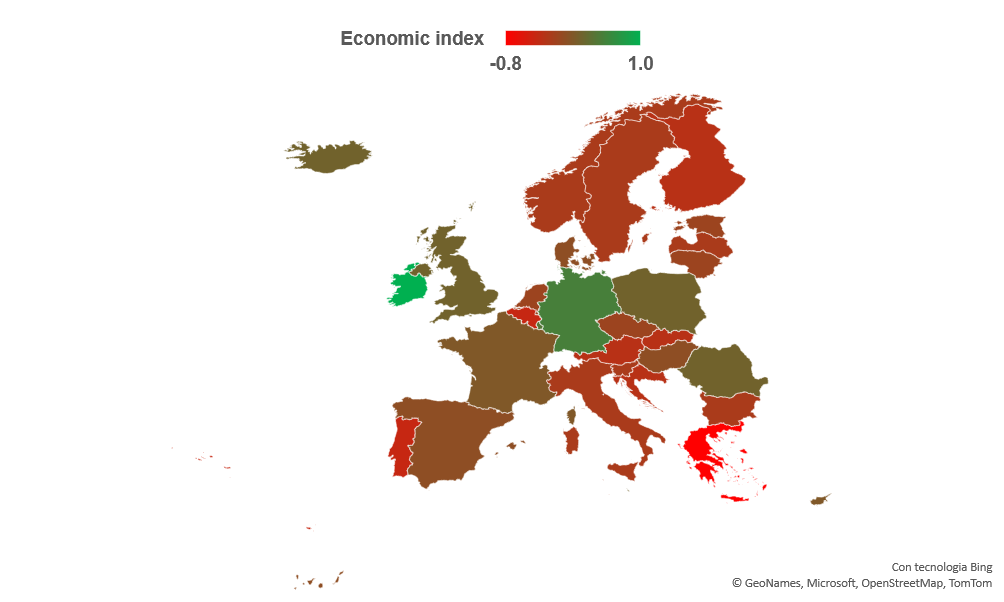


**Supplementary Figure 4. Governance index across European countries.** Map shows levels of the governance index from low (in red) to high (in green). The index was calculated as the average of standardized governance indicators (voice and accountability; political stability and absence of violence; government effectiveness; regulatory quality; rule of law; control of corruption).


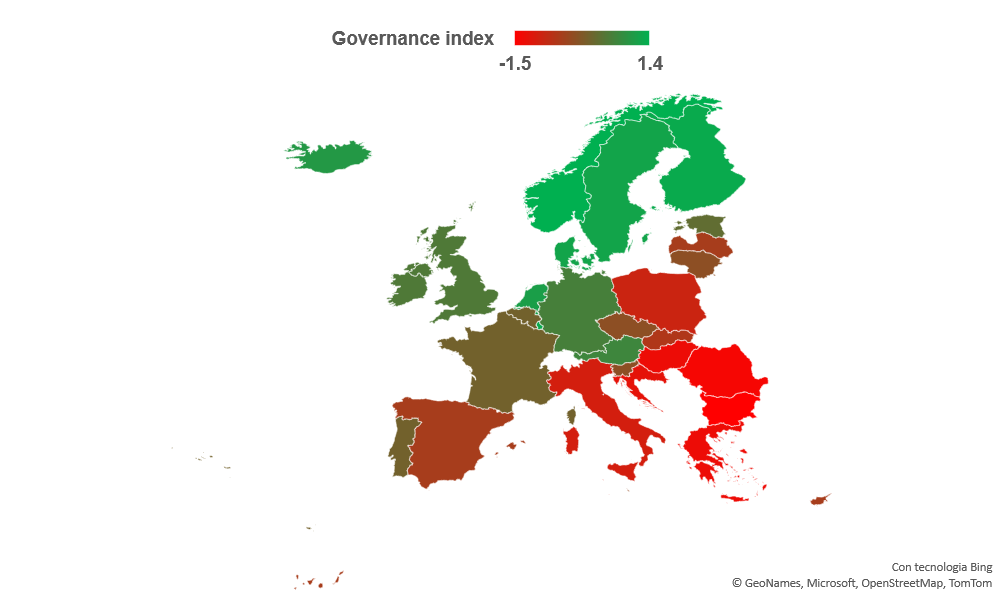


**Supplementary Figure 5. Freedom index across European countries.** Map shows levels of the freedom index from low (in red) to high (in green). The index was calculated as the average of standardized freedom and rights indicators (political rights score; civil liberties score).


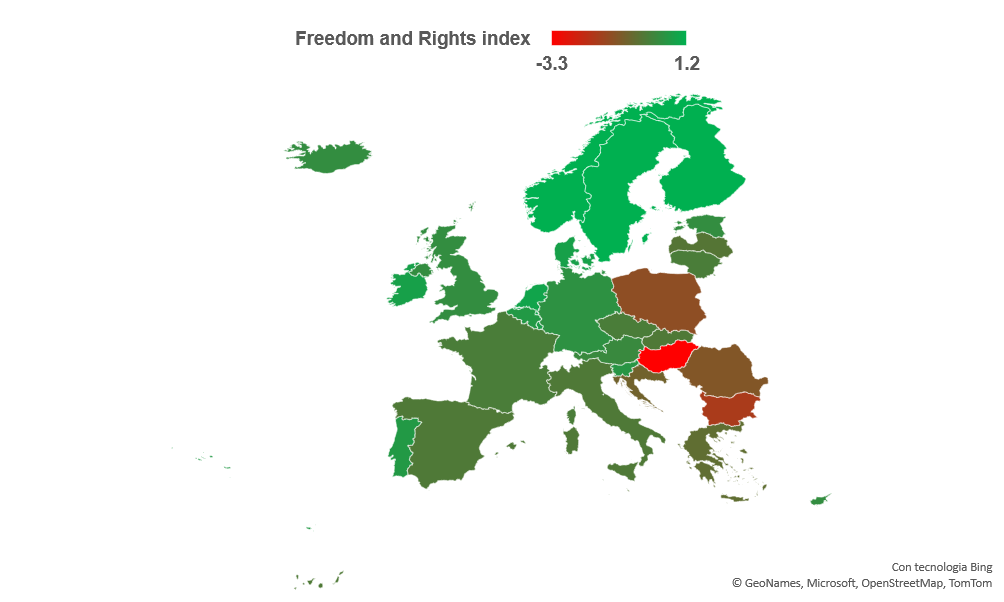

Supplement: Supplementary file 1 — Additional file 1: Table S1. Descriptive statistics of antibiotic consumption. Table S2. Spearman correlation coefficients between indicators and antibiotic consumptions. Table S3. AMR proportions for combinations between pathogens and antibiotics. Table S4. Spearman correlation coefficients between indicators and proportion of AMR for each combination between pathogen and antibiotic. Table S5. Spearman correlation coefficients between aggregate indexes and antibiotic consumption. Table S6. Differences in indexes between clusters. Table S7. Differences in antibiotic use between clusters. Figure S1. Demographic index across European countries. Figure S2. Health index across European countries. Figure S3. Economic index across European countries. Figure S4. Governance index across European countries. Figure S5. Freedom and rights index across European countries. [file 12992_2023_913_MOESM1_ESM.docx]
